# Eukaryotic Community Structure and Interspecific Interactions in a Stratified Acidic Pit Lake Water in Anhui Province

**DOI:** 10.3390/microorganisms11040979

**Published:** 2023-04-09

**Authors:** Xin Pan, Zhengbo Yue, Zhixiang She, Xiao He, Shaoping Wang, Xin Chuai, Jin Wang

**Affiliations:** 1 School of Resources and Environmental Engineering, Hefei University of Technology, Hefei 230009, China; 2 Anhui Engineering Research Center of Industrial Wastewater Treatment and Resource Recovery, Hefei 230009, China; 3 Nanshan Mining Company Ltd., Anhui Maanshan Iron and Steel Mining Resources Group, Maanshan 243000, China

**Keywords:** acid mine drainage, pit lake, eukaryotes structures, co-occurrence network, fungi-microalgae interaction

## Abstract

The stratified acidic pit lake formed by the confluence of acid mine drainage has a unique ecological niche and is a model system for extreme microbial studies. Eukaryotes are a component of the AMD community, with the main members including microalgae, fungi, and a small number of protozoa. In this study, we analyzed the structural traits and interactions of eukaryotes (primarily fungi and microalgae) in acidic pit lakes subjected to environmental gradients. Based on the findings, microalgae and fungi were found to dominate different water layers. Specifically, Chlorophyta showed dominance in the well-lit aerobic surface layer, whereas Basidiomycota was more abundant in the dark anoxic lower layer. Co-occurrence network analysis showed that reciprocal relationships between fungi and microalgae were prevalent in extremely acidic environments. Highly connected taxa within this network were *Chlamydomonadaceae*, *Sporidiobolaceae*, *Filobasidiaceae,* and unclassified Eukaryotes. Redundancy analysis (RDA) and random forest models revealed that Chlorophyta and Basidiomycota responded strongly to environmental gradients. Further analysis indicated that eukaryotic community structure was mainly determined by nutrient and metal concentrations. This study investigates the potential symbiosis between fungi and microalgae in the acidic pit lake, providing valuable insights for future eukaryotic biodiversity studies on AMD remediation.

## 1. Introduction

Acid mine drainage (AMD) represents one of the most extreme ecological environments on earth, characterized by low pH, limited nutrients, and high concentrations of sulfate and metal ions, which have led to low species richness and simple diversity in AMD ecosystems [1]. AMD has been regarded as a classical model system for microbial succession and evolution [2,3]. Remarkably, despite the harsh conditions, a broad range of acidophilic microorganisms exist in such extremely acidic environments, which are widely distributed in the three domains of life [4,5]. The significance of acidophilic microorganisms in the cycling of carbon, sulfur, iron, and nitrogen, and in geochemical processes in AMD ecosystems, has been well documented [6,7,8]. Furthermore, the study of AMD communities’ diversity has proven to be a critical topic in the field of extremophiles.

In the past few decades, the phylogenetic diversity of acidophilic archaea and bacteria inhabiting AMD has been well understood [4,9,10]. In comparison, eukaryotic communities have received much less attention. With the development of high-throughput sequencing and genomics, it is increasingly reported that eukaryotic communities in AMD ecosystems also have unexpectedly high levels of diversity and complexity [11,12,13]. The most typical example is the Rio Tinto River, where eukaryotes were the main source of biomass with a much higher species diversity than prokaryotes, with major members including microalgae, fungi, and protozoa [11,14]. Eukaryotes that survive in extremely inhospitable environments show greater resistance than prokaryotes and are actively participating in biogeochemical processes such as the decomposition and conversion of organic matter, carbon dioxide fixation, and metal accumulation and transport [7,15]. As major members of eukaryotic communities, fungi and microalgae in AMD habitats have attracted considerable interest worldwide. Fungi were well known for their high tolerance to extreme environments and their ability to biomineralize [16,17]. In addition, fungi promoted the process of carbon and nitrogen cycling in AMD with their unique degradation function [15]. Microalgae are primary producers in AMD and can use light energy to produce organic matter and oxygen, which support the growth and metabolism of heterotrophic organisms. Acidophilic or acid-tolerant microalgae, which are widely found in AMD, are also highly tolerant or resistant to low pH and toxic metals. [18,19]. As a result, exploring the diversity of eukaryotic communities goes a long way in comprehending the ecological functions and survival strategies of organisms in extreme environments.

With the progressive study of microbial communities, ecological work on AMD is shifting to microbial interactions (e.g., competition, symbiosis, parasitism, predation). Currently, interspecific interactions in extreme environments are focused on prokaryote-eukaryotes, bacteria-microalgae, and bacteria-bacteria interactions [20,21,22]. Species synergism improves the efficiency of energy and nutrient utilization. For instance, the chemoheterotrophic iron-reducing genera *Acidiphilium* can live in close symbiosis with the chemoautotrophic iron-oxidizing genera *Ferrovum*, facilitating iron cycling processes and obtaining the energy required for growth [23]. Acidophilic bacteria and microalgae can enhance metal uptake and accumulation through synergistic cooperation [21]. Earlier studies revealed symbiotic relationships between microalgae and fungi through nutrient exchange, gene signal exchange, and electron transfer [24,25,26]. However, studies on microbe-microbe interactions within eukaryotic communities in AMD are scarce. The patterns of microalgae-fungi co-occurrence in extreme environments and their responses to environmental gradients were unclear. Tracking complex microalgae-fungi interactions and designing positive symbiotic community relationships have positive implications for AMD contamination remediation [27].

This study aimed to identify the vertical distribution, co-occurrence patterns, and environmental interactions of eukaryotes in extreme ecosystems in the acidic pit lake. Based on 18S rRNA high-throughput sequencing, co-occurrence networks and correlation analysis were used to predict whether ecological interactions between fungi and algae are positive or negative and the impact of environmental factors on community interactions. We proposed the following hypotheses: (1) eukaryotic diversity in the acid pit lake exhibited a habitat-specific vertical distribution pattern and was influenced by environmental gradients; (2) more positive correlations between microalgae and fungi would help microbes adapt to extreme pressures; and (3) extreme environmental conditions in the acid pit lake can enhance the close association of microalgae and fungi.

## 2. Materials and Methods

### 2.1. Site Description and Sample Collection

In December 2019, microbial and water samples were gathered from an AMD pit lake situated in Ma’anshan City’s eastern mining region in Anhui province, China (sampling site, N 31°38′49.96″, E 118°37′35.37″) (Figure S1). The geographic locations of the sampling sites and sampling protocols are detailed in our previous studies [9]. Water was sampled at four depths (0.5 m, 2.5 m, 5 m, and 10 m) and subsequently measured for routine water quality parameters, dissolved metal ions, nutrient content, and chlorophyll a concentration detection. Simultaneously, microorganisms were collected for eukaryotic (18S) gene sequencing using a vacuum pump (SHZ-DIII, YUHUA, Shanghai, China) and 0.22 µm mixed cellulose lipid membranes, with 400–500 mL of AMD water pumped through each membrane. All samples were stored on ice at 4 °C during transportation until further processing.

For the analysis of environmental variables, water temperature (T), dissolved oxygen (DO), and pH, oxidation-reduction potential (ORP) and electrical conductivity (EC) were measured in situ on board with a portable water quality analyzer (MYRON L, 6PFC, San Diego, CA, USA) and a dissolved oxygen meter (HACH, HQ30d, Washington, USA). The concentrations of dissolved organic carbon (DOC), dissolved inorganic carbon (DIC), and total nitrogen (TN) were measured by a Multi N/C 3000 TOC analyzer (Analytik Jena AG, Jena, Thuringia, Germany). Ammonium (NH_4_^+^-N) and nitrate (NO_3_^−^-N) were determined by Nessler’s reagent spectrophotometry and ultraviolet spectrophotometry, as previously described [9]. Phosphate (PO_4_^3−^) was analyzed through ammonium molybdate spectrophotometry. Sulfate (SO_4_^2−^) was measured by ion chromatography (IC, Dionex, ICS-5000, Waltham, MA, USA). Ferrous iron (Fe(II)) was determined by phenanthroline spectrophotometry. The concentrations of total iron (TFe) and other dissolved metal ions were analyzed by ICP-MS (Agilent, 7500, Santa Clara, CA, USA). Fe(III) content is calculated from the difference between TFe and Fe(II) concentration. Chlorophyll a (Chl a) concentration was analyzed using a spectrophotometric method after extracting with 80% acetone [28].

### 2.2. Microbiome Analyses with Eukaryotic Amplicon Libraries

#### 2.2.1. Total Community DNA Extraction and Sequencing

Total microbial community DNA was extracted using FastDNA Kit for Soil (MP Biomedicals, Heidelberg, Germany) according to the manufacturer’s instructions. The quality of DNA was detected by NanoDrop™ OneC UV-Vis Spectrophotometer (Thermo, Waltham, MA, USA) to ensure the concentration and purity of the DNA. The primers 547F (5′-CCA GCA SCY GCG GTA ATT CC -3′) and V4R (5′ ACT TTC GTT CTT GAT YRA -3′) were used in the first PCR to amplify against variable region 4 (V4) of the 18S rRNA gene sequence. The PCR was performed by using a total volume of 25 μL containing 14.75 μL ultrapure water, 5 μL buffer [5×], 1 μL of each Forward and Reverse primer [10 μM], 0.25 μL Fast pfu DNA Polymerase [5 U/μL], 2 μL of dNTPs [2.5 mM], and 1 μL DNA template. The cycling program was set up with the following steps: high-temperature denaturation at 98 °C for 2 min; 20 cycles of denaturation at 95 °C, 30 s; annealing at 58 °C, 30 s; extension at 72 °C, 1 min; final extension at 72 °C, 5 min. PCR amplicons were purified with VAHTS^TM^ DNA Clean Beads (Vazyme, Nanjing, China) and quantified using the Quant-iT PicoGreen dsDNA Assay Kit (Invitrogen, Carlsbad, CA, USA). Equimolar DNA concentrations of each amplicon were then sent for paired-end Illumina MiSeq sequencing (read length: 2 × 250 bp) to Shanghai Personal Biotechnology Co., Ltd. (Shanghai, China).

#### 2.2.2. Initial Bioinformatic Analyses of 18S rRNA Gene Sequence

Analysis of sequence data using the QIIME 2 pipeline according to the tutorial provided by the QIIME developers (https://docs.qiime2.org/2023.2/tutorials/ (accessed on 1 March 2021)). Briefly, raw double-end sequencing data were imported into QIIME 2 after removing primers and barcode and quality filtering. Amplicon sequence variant (ASV) tables were generated after learning error rates, denoising, and merging paired reads using DADA2. Subsequently, the ASV feature table was annotated with species using the naive-Bayes taxonomy classifier in the qiime2-feature-classifier plugin after removing chimeras. Silva database (Release 132, http://www.arb-silva.de (accessed on 1 March 2021)). was used as the reference database at a default cutoff of 99% similarity. The raw sequence data reported in this paper have been deposited in the Genome Sequence Archive in National Genomics Data Center, China National Center for Bioinformation/Beijing Institute of Genomics, Chinese Academy of Sciences (GSA accession number: CRA009478) (https://ngdc.cncb.ac.cn/gsa (accessed on 3 February 2023)). 

### 2.3. Statistical Analysis

The ASV abundance table was rarefied at 58,248 sequences per sample. The ASV feature table was used to calculate species and distance diversity. Alpha diversity of eukaryotic communities, microalgae, and fungi was assessed by calculating multiple indices (e.g., the ASV-level richness and Shannon diversity indices) in QIIME2. To conduct statistical analysis on the alpha diversity indices of distinct groups, the “aov” and “LSD.test” functions from the “agricolae” package were employed. Microbial β-diversity with nonmetric multidimensional scaling (NMDS) based on the Bray-Curtis dissimilarity distance matrix was used to assess community similarity between different sites. We further used analysis of similarity (ANOSIM) to compare the significance of differences in microbial communities between treatments. For the pendant distribution characteristics of microalgae and fungi, the number of microbial ASV sequences in the samples was recalculated as relative abundance at the phylum and family levels, respectively. In addition, a Venn diagram was constructed to visualize unique and shared ASVs at different water depth locations.

To assess the significance of environmental factors on microbial community changes, Mantel tests based on Spearman’s correlation coefficient and permutation multivariate analysis of variance (PERMANOVA) were utilized. To further examine the relationships between eukaryotic communities at the family level and environmental gradients, redundancy analysis (RDA) and random forest (RF) models were employed. All data analysis was performed using R 4.0.5 (http://www.r-project.org/ (accessed on 5 June 2022)) and the following R packages: “vegan”, “minerva”, “reshape2”, “STATS”, and customized R scripts.

### 2.4. Co-Occurrence Networks Analysis

A co-occurrence network analysis was performed to estimate the association between microalgal and fungal communities at different water layers. The co-occurrence network is constructed as follows. First, this analysis involved constructing a network in which only ASVs with a relative abundance greater than 0.05% and detected in at least seven samples were included. Second, we calculated Spearman’s rank correlations (ρ) for all existing pairwise ASVs after screening. The eukaryotic network was built using correlations that were both robust (|ρ| > 0.7) and statistically significant (*p*-value < 0.05 after Benjamini-Hochberg FDR correction). Finally, the topological properties of the co-occurrence network, including the number of nodes, clustering coefficient, average path length, network diameter, and density were calculated to evaluate the structural characteristics of the network. The more links that are formed between nodes, the closer they are placed in the network. The size of the node in the network represents the number of connections (i.e., degree); the larger the node, the more connections it has with other species. The node attribute list and adjacent edge list were generated using R 4.0.5 with packages “Hmisc” and “igraph”. The networks were visualized by Gephi 0.9.3.

## 3. Results

### 3.1. Environmental Gradients Caused by Stratification of Pit Lakes

Table 1 summarizes the main physicochemical parameters of the water bodies collected at different locations and depths in the pit lake. The AMD pit lake was sharply stratified into an upper, oxygenated layer (0.5–2 m) and a lower anoxic reduced layer (5–10 m), as previously studied [9]. Similar to other AMD environments, the pit lake was characterized by low pH values (around 3.2–3.7), high sulfate levels, and multiple high concentrations of metal ions. Since the sampling time was winter, the water temperature of the upper water layer was slightly colder (12.35–12.70 °C) than the lake bottom (14.50–15.00 °C). Moreover, the upper water layer has lower pH and EC values but shows stronger oxidation conditions than the layers below. From 0.5 m to 10 m below the water surface, pH increased from 3.28 ± 0.03 to 3.69 ± 0.08. EC increased from 13.36 ± 0.12 mS·cm^−1^ to 22.13 ± 0.74 mS·cm^−1^. ORP decreased from 513 ±14.00 mV to 305 ± 13.00 mV.

Except for Cu and Fe(III), the concentrations of all metal ions and sulfate were significantly higher in the lower layer than in the upper layer (*p* < 0.05). In addition, surprisingly high levels of PO_4_^3−^, NH_4_^+^-N, and DIC were found in the hypolimnion, which overturned previous knowledge of oligotrophic conditions in AMD. In contrast, NO_3_-N showed the least concentration at 5 m and 10 m (0.80 mg/L). Unlike the large differences in N and P concentrations, the overall DOC content in the pit lake was low and did not show significant stratification characteristics. Eutrophic conditions corresponded to high algal biomass, and there was a gradual decrease in Chl a concentration from the upper to the lower layers.

### 3.2. Structure of Microbial Eukaryotic Communities

**Figure 1:**
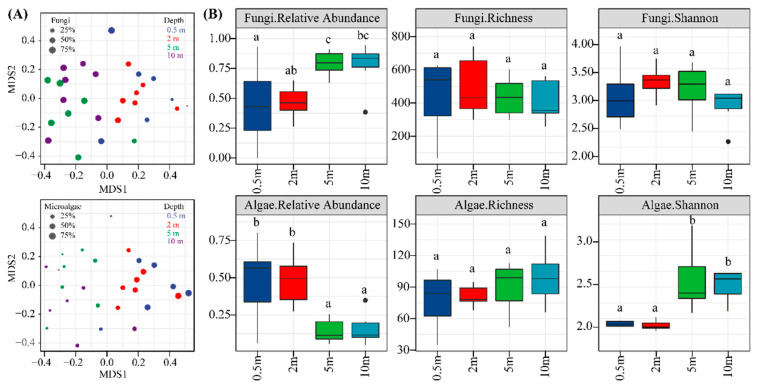
NMDS cluster analysis based on Bray-Curtis dissimilarity matrix (**A**) and alpha diversity index (**B**) for microalgae and fungi. Data with different letters represent significant differences between depths (*p* < 0.05).

Statistical analysis of NMDS based on the ANOSIM test showed that eukaryotic community composition was significantly different between surface water (0.5–2 m) and lower water (5–10 m) (R > 0.5, *p* = 0.001) (Figure 1A and Table S1). However, there were no significant differences between different sites at the same depth (R < 0, *p* > 0.1). Analysis of Venn diagrams further revealed that eukaryotic community structures were spatially heterogeneous, with a substantial number of unique ASVs detected at different depths (Figure S2). The richness, Shannon index, and Chao1 index ranged from 259 to 741, 2.28 to 3.96, and 266 to 755, respectively (Table S2). To further investigate the distribution of microalgae and fungi in different depths, we screened the ASVs representing microalgae and fungi, respectively, and recalculated the separate Richness and Shannon indices (Figure 1B). The results demonstrated that fungi had greater diversity and richness than microalgae (*p* < 0.05). Furthermore, the alpha indices of fungi and microalgae exhibited depth-dependent distribution patterns (Figure 1B). Specifically, the relative abundance of fungi increased with increasing water depth, while species richness gradually declined, and Shannon diversity showed a trend of increasing and then decreasing. Conversely, microalgae showed a higher relative abundance in the surface layer but lower species composition and richness.

**Figure 2:**
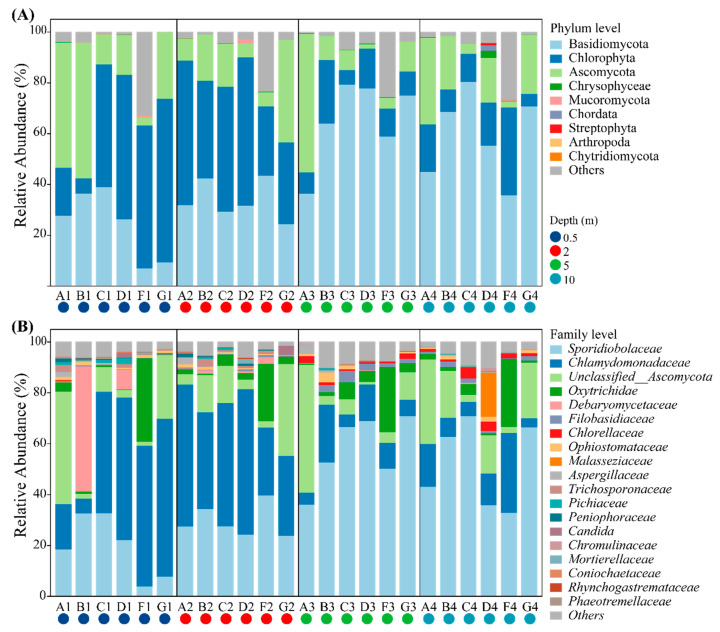
Composition of the eukaryotic community. Taxonomic assignments were conducted at phylum (**A**) and family (**B**) level.

To explore the eukaryotic community composition along the vertical differentiation in the AMD pit lake, we compared eukaryotic composition at phylum and family levels, respectively (Figure 2). Three main taxonomic groups, namely Fungi, Viridiplantae, and Ciliophoran, occupied the majority of the sequences. The analysis identified a total of 20 phyla among the three eukaryotic lineages in all samples. Chlorophyta (28.9% of the total sequences), Basidiomycota (45.09% of the total sequences) and Ascomycota (18.68% of the total sequences) were the predominant algal and fungi phylum within the eukaryotic communities. Comparing the specific composition of microalgal and fungal communities in different water layers, the Chlorophyta’s relative abundance decreased from 41.76–43.64% (upper layers) to 12.49–15.82% (lower layers) of total ASVs. In contrast, the abundance of Basidiomycota substantially increased from 24.21–33.8% to 59.2–65.17%. At the family level, Chlorophyta was composed of two distinct groups *Chlamydomonadaceae* (29.33%) and *Chlorellaceae* (0.87%), and the fungal community was mainly represented by *Sporidiobolaceae* (36.94%) and unclassified *Ascomycota* family (14.72%). Unusually, the major member of the ciliates, *Oxytrichidae*, was found only in sampling site F and had a high relative abundance (22.58–32.75%).

### 3.3. Co-Occurrence Networks of Eukaryote Communities

**Figure 3:**
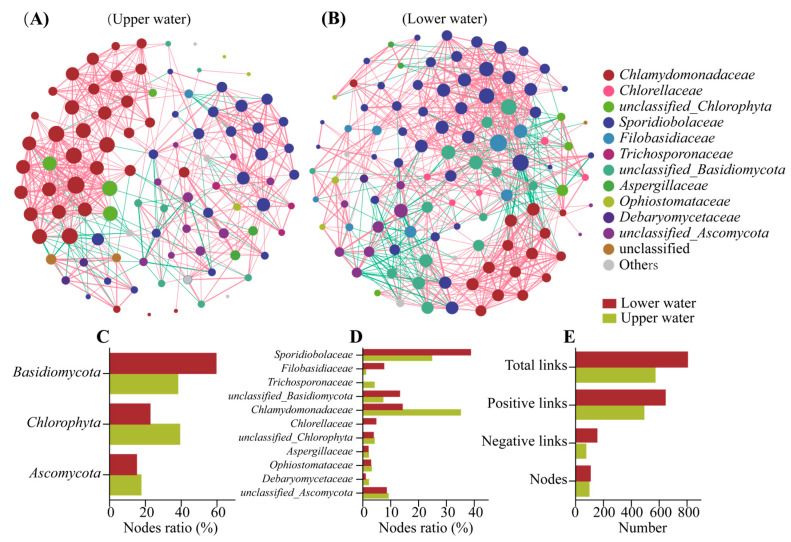
Co-occurrence network of microbial interactions based on top ASVs in the upper (**A**) and lower water (**B**). A connection stands for the significant Spearman correlation with 0.7 < |r| <1 (*p* < 0.05). The size of a node is proportional to the degree. The node color represents the assignment at family level. Number of keystones ASVs within two networks were counted at phylum (**C**) and family level (**D**). Network properties, including the number of links and nodes (**E**) were also presented.

Co-occurrence networks reveal the interactions within eukaryotic communities in different water layers of acidic pit lakes (Figure 3A,B). The network of the upper layer contained 97 nodes and 571 edges with a betweenness centralization of 0.05, while the lower layer contained 106 nodes and 804 edges with a betweenness centralization of 0.063 (Table S3). The increase in the number of edges, nodes, and betweenness centralization values indicates a stronger interaction between ASVs in the lower layer network. Topological properties are calculated to describe the complex pattern of nodes’ interrelationships (Table S3). All nodes in the networks were assigned to six eukaryotic phyla. In the upper water, nodes were mainly correlated within the phylum Basidiomycota (38.14% of node’s ratio), Chlorophyta (39.18%), and Ascomycota (17.53%) (Figure 3C). While in lower water, Basidiomycota (59.43%) became the phylum with the highest percentage of nodes, followed by Chlorophyta (22.64%), and Ascomycota (15.09%). The family identified as dominant taxa were *Sporidiobolaceae*, *Filobasidiaceae*, *Oxytrichidae*, *Ophiostomataceae*, *Chlorellaceae*, *Chlamydomonadaceae*, and unclassified Ascomycota and Basidiomycota (Figure 3D). Positive correlation links dominate ecological networks in the acid pit lake and increased from 497 in the upper layer to 644 in the lower layer. The co-occurrence network for eukaryotes throughout the acid pit lake revealed the symbiotic relationship between microalgae and fungi.

### 3.4. Relationships between Eukaryote Communities and Environmental Factors

**Figure 4:**
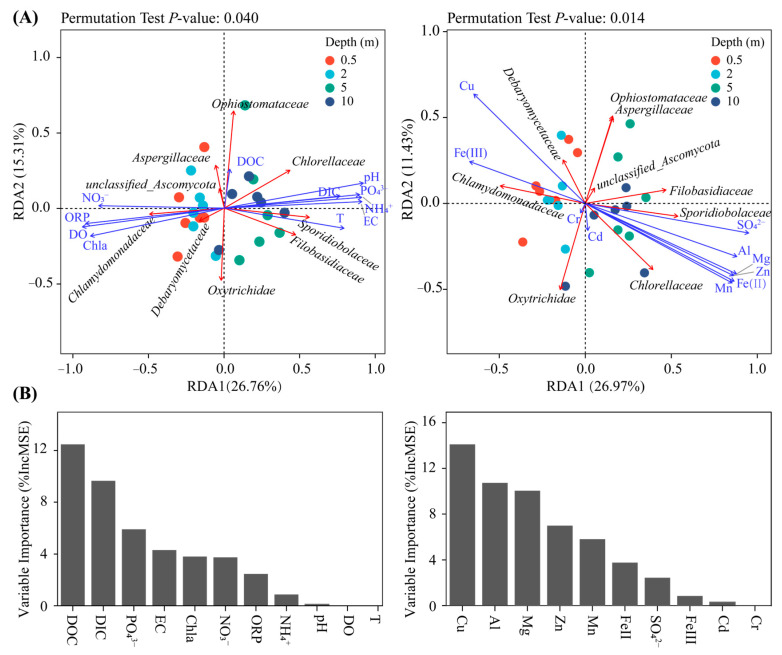
Redundancy analysis (RDA) of eukaryotic communities and environmental factors (**A**). Blue arrows with different lengths denote relative correlations of different independent variables with the biological factors. Red arrows of different lengths indicate different content of family level groups. Random Forest model output relating geochemical parameters to eukaryotic community richness in four depths (**B**).

The Mantel test revealed that environmental factors had a stronger correlation with variation in Chlorophyta and Basidiomycota than with Ascomycota (Table S4). Different environmental variables have different degrees of contribution to shaping the community structure. RDA analysis and RF models were used to further assess and quantify the effects of environmental parameters on the relative abundance distribution of family taxa and observed species number (Figure 4A,B). *Chlamydomonadaceae* was positively correlated with Chl a, DO, NO_3_^−^, Fe(III), and Cu. Meanwhile, *Sporidiobolaceae*, *Filobasidiaceae,* and *Chlorellaceae* were more closely related to T, pH, PO_4_^3−^, sulfate, and metal ions enriched in the lower layers. Although DOC had a less pronounced effect on the relative abundance of taxa, it significantly contributed to community diversity. Among the environmental variables evaluated, DOC, DIC, PO_4_^3−^, EC, Cu, Al, Mg, and Zn were revealed to have the greatest impact on community diversity.

## 4. Discussion

To date, few studies have explored eukaryotic diversity and intra-community interactions in AMD pit lakes. In this study, we focus on an acidic pit lake with sharp stratification, low pH, and high levels of toxic metals and sulfates. A comprehensive survey of eukaryotes was conducted in combination with 18S rRNA high-throughput sequencing, water chemistry analysis, and statistical methods to characterize the microbial responses to environmental gradients in the pit lake. Based on these findings, the water column teemed with indigenous microalgae and fungi, but their vertical distribution varied in terms of community composition, diversity, and coexistence patterns. Uncovering the interactions between “microalgae-fungi” and “eukaryote-environment” will facilitate our future design of these microorganisms for AMD remediation.

### 4.1. Spatial Heterogeneity and Ecological Role of Eukaryotic Communities

We investigated the eukaryotic community structure of AMD samples from four depths to understand the spatial heterogeneity and ecological roles of microalgae and fungi. According to the results of α-diversity analysis and taxonomic composition, microalgae and fungi dominated the surface and bottom of the lake, respectively. The abundance of Chlorophyta in the surface layer is caused by intense light, sufficient atmospheric inorganic carbon, and adequate levels of nitrogen and phosphorus nutrients for algal growth. This is consistent with findings from the Rio Tinto River in Spain, where the eukaryotic community also consists mainly of photosynthetic algae [11]. The main member of Chlorophyta is *Chlamydomonadaceae*, a flagellated unicellular alga, which is a common acidophilic green alga in AMD [29]. Moreover, *Chlamydomonadaceae* has shown a good tolerance to a wide range of physical and chemical conditions in AMD-contaminated lakes and maintains its presence throughout the process [30]. Green algae, as primary producers in the pit lake environment, play a crucial ecological role. Photosynthesis in acidophilic algae generates oxygen, which improves the biological oxidation of ferrous iron, reduced inorganic sulfur compounds, and low valent arsenic. Moreover, the organic matter excreted by these algae provides crucial nutrients for the growth and metabolism of heterotrophic organisms [31]. Additionally, surface blooms of algal biomass have the ability to take up and immobilize assimilated metals in sediment through deposition, ultimately reducing the biotoxicity of AMD [32].

Fungi are another important member of the eukaryotic community in the AMD habitat. Existing studies have demonstrated that fungi have a wide range of pH tolerance, such as *Aspergillus* and *Penicillium* species that can survive at pH 2 [33]. In this study, Basidiomycota and Ascomycota exhibited high proportions at all sampling depths (Figure 2A). The degradation process of complex organic matter, such as leaves and dead wood, in the acidic pit lake is severely limited due to the absence of invertebrates. In this context, saprophytic trophic fungi can function as primary decomposers of organic matter, and therefore, they play a pivotal role in the carbon and nitrogen cycle of the system. This assertion is backed by our previous research on the composition of dissolved organic matter in the pit lake. We found that DOM in the lower water column is mainly comprised of fulvic-like substances and aromatic compounds, which are challenging for organisms to use directly [34,35]. Ascomycota and Basidiomycota fungi have drawn significant attention for their biomineralization ability and their capacity for intracellular enrichment of toxic heavy metals. Analysis of the fungal sequences in this study indicates that the most abundant ASV belongs to the *Rhodotorula mucilaginosa* (*Sporidiobolaceae*) (Figure S3). This fungus can accumulate Zn and Pb through biosorption processes and excrete the siderophore compound, which is strongly associated with metal resistance [36]. Some species with less metal tolerance can still withstand high-stress environments by employing similar mechanisms to remove toxic metals [37]. *Rhodotorula* species, known as inhabitants of cold environments, may have growth advantages in winter [38,39]. Additionally, *Rhodotorula* may enhance algal growth through the production of rhodotorulic acid, which has plant growth-promoting properties through potential microalgae-fungi interactions. [40].

Except for fungi and microalgae, a large number of *Oxytrichidae* were found in the area of sampling site F. Protozoa and could affect the microbial population and biomass in AMD pit lake by preying on Chlorophyta, bacteria, and archaea [41]. The special community composition of site F may be due to the sampling location near the densely vegetated southern shore, which is susceptible to terrestrial sources of organisms and organic matter.

### 4.2. Network Analysis Reveals Co-Occurrence Patterns in Eukaryotes

The structure of the co-occurrence network provides new insight into the internal relationships of eukaryotic communities. Within these communities, microbial interactions can be classified as either positive or negative, representing mutually-beneficial or mutually-exclusive relationships, respectively. Intra-domain co-occurrence network analysis revealed a predominantly positive correlation between algae and fungi in the acid pit lake, indicating a strong ecological relationship or ecological niche sharing among microorganisms. Depending on the proportions and interrelationships of *Sporidiobolaceae*, *Filobasidiaceae*, *Chlamydomonadaceae,* and *Chlorellaceae* in the molecular network, it was determined that algae and fungi dominated the upper and lower symbiotic networks, respectively (Figure 3C–E). The large number of flagellated acidophilic *Chlamydomonadaceae* aggregated in the upper layer will create a unique algal microenvironment, which can provide habitat and nutrition for heterotrophic fungi [42]. Accordingly, the algae can absorb CO_2_ produced by the respiration of fungi such as *Sporidiobolaceae* and *Filobasidiaceae*, preventing carbon and other nutrients from being lost from the environment.

In extreme environmental conditions, there is evidence to suggest that fungal and microalgal symbiosis may become more prevalent. In the lower water (5–10 m), where metal and sulfate concentrations are higher and dissolved oxygen content is lower, the network nodes were more diversified and had more positive links. Synergistic symbiosis of microalgae-fungi may be an important survival strategy for microorganisms to cope with extreme environments. Previous studies in the Yudong River basin in Guizhou, China, also showed an increase in positive interactions between members of the microbiome contaminated by AMD compared to uncontaminated areas [43]. Microalgae and fungi may co-create a microenvironment with neutral pH and low heavy metal concentration through functional and morphological complementarities in order to withstand the stress of extreme environments [20]. In AMD ecosystems, this microenvironment is usually in the form of biofilms, through which algae and fungi are able to exchange nutrients, transfer genes and transmit signals, etc. [44]. The emergence of biofilms is indispensable for microalgae-fungi symbiosis, as the formation of structured biofilms protects cells from external stressful conditions and allows them to resist more extreme environments [45,46]. Comparing the topological characteristics and node proportions of different pelagic networks, fungi may play a more important ecological function in the anaerobic hypolimnion. *Sporidiobolaceae* are the most connected nodes in the lower network, and its important member *Rhodotorula* was shown to be able to form a symbiotic system with various microalgae. Martin et al. discussed that *Rhodotorula* can form a positive symbiosis with various algae, including *Chlorella*, *Chlamydomonas,* and *Desmodesmus* [25]. It was also observed that the co-culture system of microalgae and *Rhodotorula* could increase biomass and produce large amounts of lipids [25]. The smaller *Rhodotorula* in the symbiotic structure may be tightly attached to the flagellum of *Chlamydomonas* or the surface of *Chlorella*, greatly increasing the efficiency of nutrient utilization and pollutant removal [47].

Except for the positive interaction between microalgae and fungi, there were mainly negative correlations among different microalgae groups, indicating that there may be competition or predation between taxa. In conditions where resources are scarce, for example in this pit lake, *Chlamydomonadaceae* and *Chlorellaceae* compete for limited nutrient elements such as nitrogen, phosphorus, and light. This competition conforms to the principle of competitive exclusion, which asserts that similar species cannot coexist in the same ecological niche [48]. Among these species, *Chlamydomonadaceae* has been observed to have a competitive advantage in winter due to its high tolerance for colder climates. It is crucial to acknowledge that not all microalgae-fungi associations in AMD can be characterized by correlation methods alone. These methods are not always reliable, as they may not necessarily reflect the actual association between populations and can also be influenced by external environmental factors. Although these correlations do provide a partial understanding of the potential interactions that may occur, it is important to supplement these findings with co-culture experiments to establish more specific relationships between microalgae and fungi in AMD. Consequently, future studies should focus on undertaking these co-culture experiments to provide a more comprehensive understanding of the associations between algae and fungi in AMD.

### 4.3. Response of Fungi and Green Algae to Environmental Gradients

The abundance of algae biomass in the pit lake is closely related to the eutrophication degree of water. Algal distribution is affected mainly by the limited availability of bioavailable nitrogen and phosphate content. RDA analysis showed that NH_4_^+^ and NO_3_^−^ were significantly negatively correlated with *Chlamydomonadaceae* and *Chlorellaceae*, respectively, which was consistent with the preference of algae for different nitrogen sources: the former preferred NH_4_^+^, while the latter could utilize both types of nitrogen (Figure 4A). This also explains the lower levels of ammonia in the surface water. Additionally, the assimilation process of ammonia by algae releases H^+^ into the medium, while the assimilation process of nitrate produces OH^−^, indirectly contributing to the pH stratification of the pit lake [49]. Consistent with ammonia nitrogen, DIC, PO_4_^3−^, and other substances closely related to the growth and metabolism of microalgae were negatively correlated with the abundance of *Chlamydomonadaceae*. Kumar et al. discovered that the primary limiting factor for microalgae growth in acid pit lakes is the low concentration of nutrient elements, particularly phosphorus, instead of high metal ion concentrations or low pH levels in their study [50]. The pit lake’s DIC can be attributed solely to CO_2_, given the negligible concentration of bicarbonate due to the low pH value. Previous studies have established the significant influence of CO_2_ concentration on the growth of acidophilic Chlamydomonas, with low CO_2_ levels contributing to increased primary productivity and photosynthetic rates [51]. Additionally, Chlamydomonadaceae utilize DIC to synthesize organic matter that is subsequently transported to heterotrophic organisms for further processing [30]. Conversely, an increase in fungal abundance exacerbates the conversion of DOC to DIC, leading to the accumulation of higher concentrations of CO_2_ in the underlying water. Low DO levels reflect substantial respiratory activity among lower-layer heterotrophs, with eukaryotic communities dominated by mixotrophic and heterotrophic fungi. As a result of rapid heterotroph depletion, DOC levels within the pit lake remain persistently low.

The distribution of fungi and microalgae was impacted by ORP, DO, pH, EC, and Chl a to varying degrees, as shown in Figure 4B. Among these factors, chlorophyll a is an important indicator of algae biomass in aquatic ecosystems, with photosynthesis producing oxygen as the primary product. Both factors are significantly positively correlated with the abundance of Chlamydomonadaceae. Additionally, ORP plays a key role in the stratification of acidic pit lake communities, with its value determining the distribution of aerobic/anaerobic taxa. pH has been identified as the most influential factor shaping the AMD bacterial community, though its effect on eukaryotes was not as pronounced in this study [52]. Compared with the strong tolerance of fungi to low pH, microalgae are more sensitive to pH changes. Under extremely acidic conditions, weak pH fluctuations may greatly affect the succession of microalgae.

Microalgae and fungi living in extremely acidic environments have evolved unique adaptations to low pH and heavy metals. Aguilera et al. have also found that the diversity of indigenous algae growing in biofilms is related to the acidity and metal content of the environment [53]. Mantel test results showed that in the pit lake, high levels of metal cations and sulfate anions were crucial environmental factors in discriminating between dominant species (Table S4). Figure 4A indicates that apart from Cu and Fe^3+^, other metal ions and sulfate are significantly negatively correlated with *Chlamydomonadaceae*. On the one hand, the biological toxicity of metal ions to algae is apparent, with excessive copper proving fatal to the photosynthetic algae by inhibiting green algae’s photosynthetic activity and chloroplast degradation. In a similar vein, a high concentration of ferrous iron causes *Chlamydomonas reinhardtii* to have chlorosis and reduced growth activity [54]. High metal concentrations can also indirectly affect community structure by inhibiting nutrient absorption and utilization in microorganisms and forming complex precipitates with essential nutrients [54]. Therefore, algae abundance was often negatively correlated with a dissolved metal concentration in AMD water [55]. Compared with other algae, *Chlamydomonadaceae* has a higher tolerance to Cu, which also gives it an advantage in the internal competition of surface green algae [29]. Multiple fungal families (e.g., *Sporidiobolaceae*, *Filobasidiaceae*) showed significant positive correlations with metal concentrations, which is not surprising, as fungi are much more tolerant to metal ions than algae. On another note, the ability of algae and fungi in AMD habitats to accumulate metals and other contaminants has attracted considerable attention [56]. The free metal ions can combine with exopolysaccharides released by the cell wall/membrane of eukaryotes and move to the lower water layers’ sedimentation with the release of cell lysis [37,57]. Aguilera et al. also found that the Rio Tinto biofilm composed of eukaryotes could precipitate heavy metals in the water via the complexation of extracellular polymers with heavy metals [32]. This ability to accumulate metals and other contaminants gives eukaryotes great biotech potential in AMD remediation.

## 5. Conclusions

The study analyzed the eukaryotic community composition and its correlation with environmental factors in an acidic mining pit lake located in Anhui Province, China. The findings indicate that the distribution and composition of eukaryotic communities in the pit lake had obvious vertical stratification characteristics. Green algae and fungi were dominant in the surface layer and the bottom layer, respectively, which were mainly represented by *Chlamydomonadaceae* and *Sporidiobolaceae*. By constructing the co-occurrence networks, we have identified the interactions of eukaryotic communities and revealed the potential mutually-beneficial symbiotic relationship between microalgae and fungi. The next step should be to verify the specific relationship between them through experiments. RDA analysis showed that water quality was the main factor determining the composition and diversity of microeukaryote communities. The sharp increase in metal concentration and the decrease in DO promote communication and cooperation between species. Similarly, changes in community structure in turn affect the spatial differences in the physical and chemical properties of the pit lake. These results provide preliminary information for understanding the eukaryotic community composition and population relationship in acid pit lakes and provide a theoretical basis for the development of bioremediation technology in acid mining pit lakes.

## Figures and Tables

**Table 1:** Physicochemical parameters (mean ± SD) of AMD water at four depths in the pit lake.

Sampling Depth	0.5 m	2 m	5 m	10 m
T (°C) (±SD)	12.35 (±0.90)	12.70 (±0.59)	14.50 (±0.56)	15.00 (±0.88)
pH (±SD)	3.28 (±0.03)	3.27 (±0.05)	3.67 (±0.06)	3.69 (±0.08)
DO (mg L^−1^) (±SD)	14.52 (±0.43)	13.60 (±0.82)	3.84 (±1.23)	2.03 (±0.69)
ORP (mV) (±SD)	513 (±14.00)	528 (±5.00)	320 (±10.00)	305 (±13.00)
EC (ms cm^−1^) (±SD)	13.36 (±0.12)	13.34 (±0.07)	21.62 (±1.13)	22.13 (±0.74)
DOC (mg L^−1^) (±SD)	3.64 (±0.94)	3.67 (±0.53)	3.52 (±0.80)	3.06 (±1.14)
DIC (mg L^−1^) (±SD)	0.00 (±0.00)	0.00 (±0.00)	20.42 (±6.08)	22.76 (±5.19)
TN (mg L^−1^) (±SD)	5.82 (±0.43)	5.76 (±0.27)	15.69 (±0.93)	15.34 (±1.21)
NO_3_^−^-N (mg L^−1^) (±SD)	1.55 (±0.04)	1.67 (±0.17)	0.80 (±0.29)	0.80 (±0.19)
NH_4_^+^-N (mg L^−1^) (±SD)	5.61 (±1.78)	6.20 (±1.15)	14.13 (±2.84)	14.6 (±2.30)
PO_4_^3−^ (mg L^−1^) (±SD)	1.65 (±0.13)	1.58 (±0.08)	21.23 (±3.26)	24.22 (±3.24)
Chl a (μg L^−1^) (±SD)	74.28 (±18.11)	59.51 (±16.07)	12.05 (±2.45)	8.16 (±2.51)
SO_4_^2−^ (mg L^−1^) (±SD)	16789 (±911)	15913 (±1933)	28144 (±2446)	27490 (±3463)
Mg (mg L^−1^) (±SD)	1974 (±106)	1989 (±116)	4291 (±305)	4446 (±271)
Al (mg L^−1^) (±SD)	692 (±33)	694 (±37)	867 (±26)	898 (±45)
Mn (mg L^−1^) (±SD)	353 (±9)	358 (±10)	707 (±54)	743 (±26)
TFe (mg L^−1^) (±SD)	90.5 (±2.52)	90.92 (±3.31)	457 (±53)	499 (±21)
Fe(II) (mg L^−1^) (±SD)	18.16 (±1.10)	18.23 (±1.04)	409 (±45)	448 (±27)
Fe(III) (mg L^−1^) (±SD)	72.35 (±2.14)	72.68 (±3.01)	47.67 (±14.21)	50.88 (±11.50)
Cu (mg L^−1^) (±SD)	32.38 (±0.87)	32.48 (±1.02)	29.61 (±0.67)	28.86 (±0.96)
Zn (mg L^−1^) (±SD)	18.71 (±0.49)	18.77 (±0.56)	31.75 (±1.94)	32.88 (±1.14)
Cr (mg L^−1^) (±SD)	0.027 (±0.001)	0.026 (±0.002)	0.036 (±0.002)	0.038 (±0.002)
Cd (mg L^−1^) (±SD)	0.148 (±0.002)	0.149 (±0.002)	0.233 (±0.016)	0.239 (±0.005)

## Data Availability

The raw sequence data reported in this paper have been deposited in the Genome Sequence Archive (Genomics, Proteomics & Bioinformatics 2021) in National Genomics Data Center (Nucleic Acids Res 2022), China National Center for Bioinformation/Beijing Institute of Genomics, Chinese Academy of Sciences (GSA: CRA009478) and are publicly accessible at https://ngdc.cncb.ac.cn/gsa.r (accessed on 3 February 2023).

## Supplementary Materials

The following supporting information can be downloaded at: https://www.mdpi.com/article/10.3390/microorganisms11040979/s1, Figure S1: Schematic diagram of the geographic location of the sampling sites. Figure S2: Veen diagram showing the number of ASVs common and unique to the four depth samples. Figure S3: Abundance distribution of the top 15 eukaryotic species in acidic pit lake. Table S1: Analysis of intergroup variation in eukaryotic communities at different water depths; Table S2: Eukaryotic community alpha diversity indices; Table S3: Topological properties of co-occurring networks; Table S4: Simple Mantel tests for correlations between environmental factors (Euclidean distance) and microeukaryotes communities with 999 permutations.
