# Supplementary material for: Eukaryotic Community Structure and Interspecific Interactions in a Stratified Acidic Pit Lake Water in Anhui Province
Source: Microorganisms. 2023 Apr 9;11(4):979. doi: 10.3390/microorganisms11040979 (PMC10142529; doi:10.3390/microorganisms11040979)
Supplement: Supplementary file 1 [file microorganisms-11-00979-s001.zip › microorganisms-2288665-supplementary.pdf]

Supplementary Material

Eukaryotic community structure and interspecific interactions in a stratified acidic pit lake in Anhui Province

Pan Xin[1,2], Yue Zhengbo[1,2*], She Zhixiang[1,2], He Xiao[3], Wang Shaoping[3], Chuai Xin[3], Wang Jin[1,2*]

[1] School of Resources and Environmental Engineering, Hefei University of Technology, Hefei, Anhui 230009, China
[2] Key Laboratory of Nanominerals and Pollution Control of Anhui Higher Education Institutes, Hefei University of Technology, Hefei, Anhui 230009, China
[3] Nanshan Mining Company Ltd, Anhui Maanshan Iron and Steel Mining Resources Group, Maanshan, Anhui, 243000, China
* Correspondence: sophiawj@hfut.edu.cn; zbyue@hfut.edu.cn; Tel: 86-551-62901523; Fax: 86-551-62901524.

1 Supplementary Figures and Tables

1.1 Supplementary Tables

Table S1. Analysis of intergroup variation in eukaryotic communities at different water depths

Group1	Group2	Permutations	R	p-value	q-value
all	-	999	0.381	0.001	-
0.5 m	2 m	999	-0.034	0.628	0.754
0.5 m	5 m	999	0.540	0.002	0.003
0.5 m	10 m	999	0.586	0.001	0.002
2 m	5 m	999	0.631	0.001	0.002
2 m	10 m	999	0.697	0.001	0.002
5 m	10 m	999	-0.089	0.836	0.836

Table S2. Eukaryotic community alpha diversity indices

		Richness	Chao1	ACE	Shannon	Simpson	Coverage	PD
0.5 m	A	627	642	651	3.96	0.94	0.9992	13.71
	B	617	626	635	2.91	0.79	0.9994	10.37
	C	540	562	570	3.08	0.85	0.9992	10.84
	D	607	625	637	3.36	0.88	0.9992	9.85
	F	334	339	344	2.49	0.80	0.9997	27.02
	G	311	324	324	2.64	0.82	0.9997	7.12
2 m	A	657	671	686	3.47	0.88	0.9991	15.93
	B	652	680	687	3.75	0.93	0.9990	10.22
	C	432	459	457	3.35	0.90	0.9993	9.75
	D	741	756	769	3.38	0.86	0.9991	16.22
	F	424	434	439	2.92	0.87	0.9995	15.42
	G	300	307	309	3.17	0.89	0.9997	7.00
5 m	A	321	345	342	2.44	0.74	0.9995	11.32
	B	601	629	633	3.68	0.90	0.9992	14.27
	C	524	536	540	3.59	0.91	0.9995	16.16
	D	511	518	524	3.33	0.87	0.9996	12.36
	F	298	310	313	2.93	0.88	0.9997	12.76
	G	434	443	445	3.26	0.89	0.9996	15.32
10 m	A	334	345	349	2.81	0.85	0.9995	14.99
	B	525	543	552	3.08	0.80	0.9993	23.42
	C	544	565	576	3.59	0.92	0.9992	13.71
	D	560	571	578	3.12	0.88	0.9998	18.70
	F	259	266	267	2.28	0.80	0.9998	10.82
	G	355	372	371	3.00	0.87	0.9996	9.35

Table S3 Topological properties of co-occurring networks

Topological properties	Upper network	Lower network
Nodes	97	571
Edges	106	804
Clustering coefficient (CC)	0.633	0.602
Average degree (AD)	11.733	15.170
Network diameter (ND)	6	5
Graph density (GD)	0.123	0.144
Betweenness centralization	0.050	0.063
Degree centralization	0.138	0.160
Modularity (MD)	0.62	0.534
Average path length (APL)	2.725	2.394

Table S4. Simple Mantel tests for correlations between environmental factors (Euclidean distance) and microeukaryotes communities with 999 permutations

env	Chlorophyta		Basidiomycota		Ascomycota	
	r.value	p.value	r.value	p.value	r.value	p.value
Depth	0.293	0.002	0.388	0.001	0.016	0.394
T	0.268	0.003	0.445	0.001	-0.017	0.519
pH	0.431	0.001	0.456	0.001	-0.036	0.685
DO	0.466	0.001	0.466	0.001	0.031	0.221
ORP	0.428	0.002	0.580	0.001	-0.024	0.632
EC	0.440	0.001	0.541	0.001	0.061	0.154
PO_4^{3-}	0.471	0.001	0.520	0.001	0.000	0.439
DOC	0.016	0.358	-0.063	0.720	-0.044	0.591
DIC	0.372	0.001	0.457	0.001	0.081	0.137
TN	0.333	0.003	0.399	0.001	0.050	0.231
NO_3^--N	0.433	0.001	0.305	0.001	-0.073	0.865
NH_4^+-N	0.301	0.005	0.403	0.001	-0.019	0.525
Chl a	0.403	0.002	0.545	0.001	-0.033	0.622
SO_4^{2-}	0.556	0.001	0.378	0.001	-0.033	0.694
Mg	0.452	0.001	0.578	0.001	0.009	0.376
Al	0.460	0.002	0.602	0.001	-0.015	0.564
Mn	0.398	0.002	0.518	0.001	0.050	0.198
TFe	0.401	0.001	0.524	0.001	0.032	0.270
Fe(II)	0.449	0.001	0.524	0.001	0.028	0.303
Fe(III)	0.153	0.026	0.238	0.006	0.032	0.319
Cu	0.284	0.003	0.497	0.001	-0.067	0.778
Zn	0.433	0.001	0.461	0.001	0.029	0.273
Cr	0.464	0.001	0.550	0.001	-0.055	0.849
Cd	0.391	0.001	0.441	0.001	0.057	0.182

1.2 Supplementary Figures

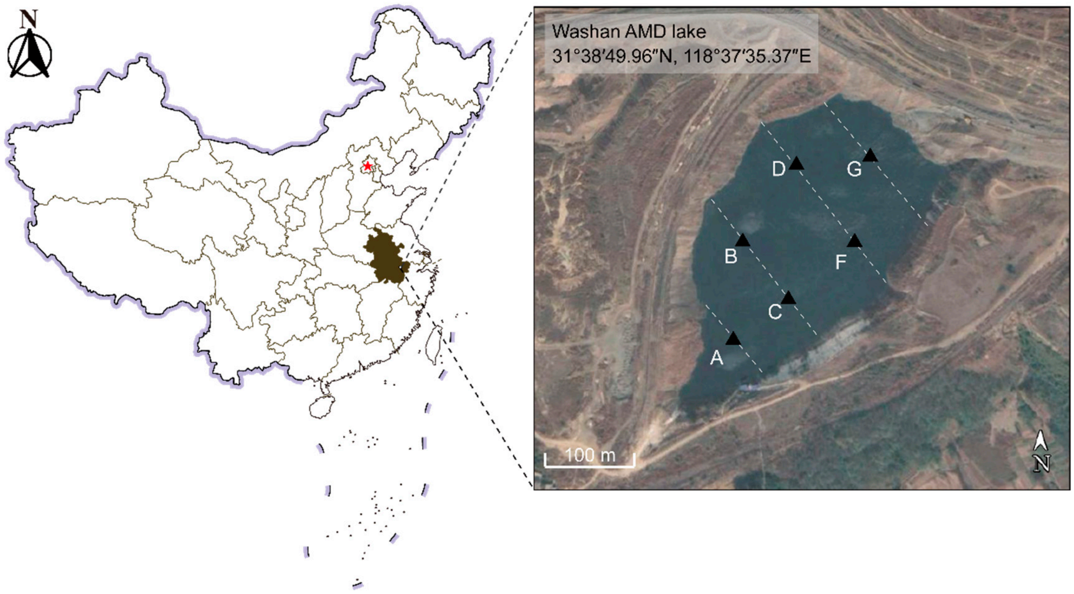

Figure S1. Schematic diagram of the geographic location of the sampling sites. (A, B, C, D, F, G A, B, C, D, F, G represent the different sampling locations)

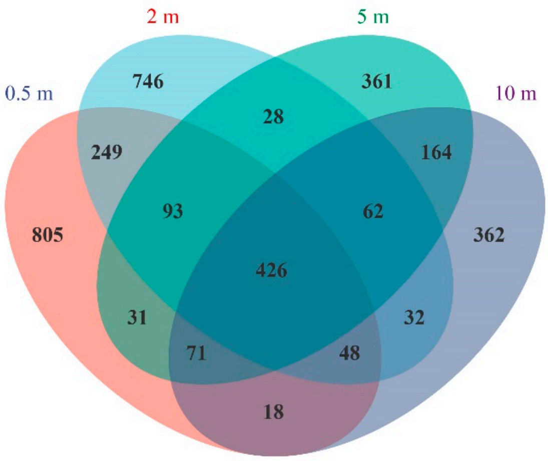

Figure S2. Veen diagram showing the number of ASVs common and unique to the four depth samples.

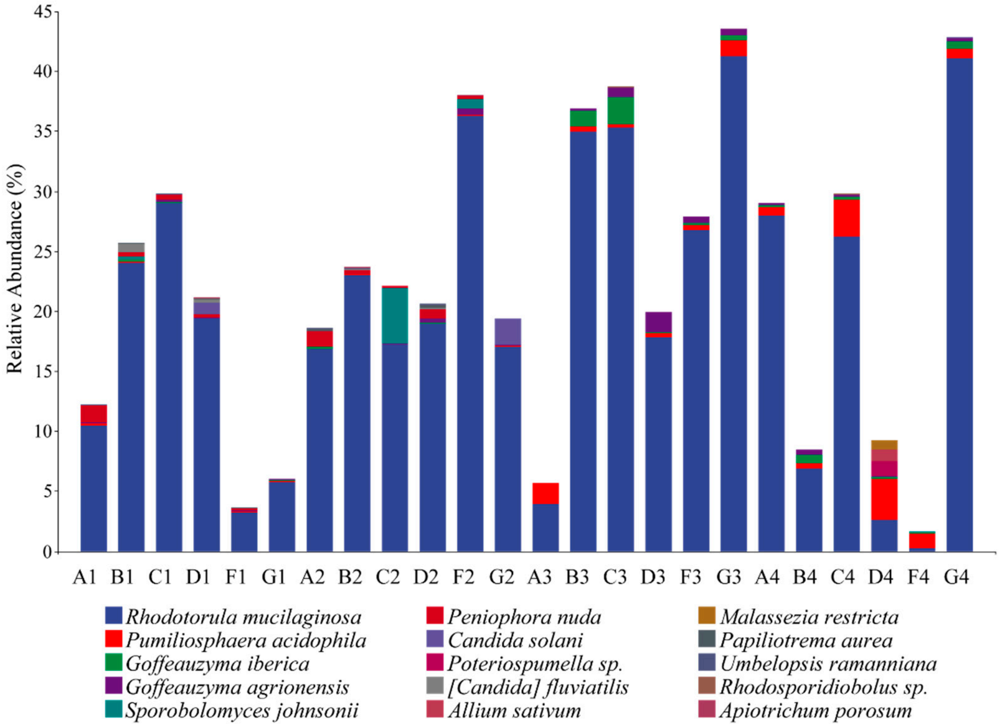

Figure S3. Abundance distribution of the top 15 eukaryotic species in acidic pit lake.
